# Large Scale Screening of Digeneans for *Neorickettsia* Endosymbionts Using Real-Time PCR Reveals New *Neorickettsia* Genotypes, Host Associations and Geographic Records

**DOI:** 10.1371/journal.pone.0098453

**Published:** 2014-06-09

**Authors:** Stephen E. Greiman, Vasyl V. Tkach, Eric Pulis, Thomas J. Fayton, Stephen S. Curran

**Affiliations:** 1 Department of Biology, University of North Dakota, Grand Forks, North Dakota, United States of America; 2 Department of Coastal Sciences, The University of Southern Mississippi, Ocean Springs, Mississippi, United States of America; Auburn University, United States of America

## Abstract

Digeneans are endoparasitic flatworms with complex life cycles including one or two intermediate hosts (first of which is always a mollusk) and a vertebrate definitive host. Digeneans may harbor intracellular endosymbiotic bacteria belonging to the genus *Neorickettsia* (order Rickettsiales, family Anaplasmataceae). Some *Neorickettsia* are able to invade cells of the digenean's vertebrate host and are known to cause diseases of wildlife and humans. In this study we report the results of screening 771 digenean samples for *Neorickettsia* collected from various vertebrates in terrestrial, freshwater, brackish, and marine habitats in the United States, China and Australia. *Neorickettsia* were detected using a newly designed real-time PCR protocol targeting a 152 bp fragment of the heat shock protein coding gene, GroEL, and verified with nested PCR and sequencing of a 1371 bp long region of 16S rRNA. Eight isolates of *Neorickettsia* have been obtained. Sequence comparison and phylogenetic analysis demonstrated that 7 of these isolates, provisionally named *Neorickettsia* sp. 1–7 (obtained from allocreadiid *Crepidostomum affine*, haploporids *Saccocoelioides beauforti* and *Saccocoelioides lizae*, faustulid *Bacciger sprenti*, deropegid *Deropegus aspina*, a lecithodendriid, and a pleurogenid) represent new genotypes and one (obtained from *Metagonimoides oregonensis*) was identical to a published sequence of *Neorickettsia* known as SF agent. All digenean species reported in this study represent new host records. Three of the 6 digenean families (Haploporidae, Pleurogenidae, and Faustulidae) are also reported for the first time as hosts of *Neorickettsia*. We have detected *Neorickettsia* in digeneans from China and Australia for the first time based on PCR and sequencing evidence. Our findings suggest that further surveys from broader geographic regions and wider selection of digenean taxa are likely to reveal new *Neorickettsia* lineages as well as new digenean host associations.

## Introduction

*Neorickettsia* (family Anaplasmataceae) is a genus containing obligate intracellular endosymbionts of digeneans (Platyhelminthes, Digenea). Although relatively small, this genus has received significant attention in the recent years, with a rapid increase in the number of known species level lineages in this group [1], [2], [3], [4], [5]. *Neorickettsia* are vertically transmitted through all stages of complex digenean life cycles. Additionally, they are capable of being horizontally transmitted to the vertebrate hosts of the digenean, both human and wildlife, where they can cause disease [1], [4], [6], [7], [8], [9]. These diseases are potentially debilitating, e.g., Sennetsu fever in humans (*Neorickettsia sennetsu*), or even fatal, e.g., salmon dog poisoning (*Neorickettsia helminthoeca*), and Potomac horse fever (*Neorickettsia risticii*) [4].

*Neorickettsia* have now been reported from several countries and all continents including Antarctica [4], [5]. However, a majority of the records are based almost exclusively on immunological detection of *Neorickettsia* in vertebrate hosts, mainly horses. It should be noted that positive immunological tests of horses could be a result of either actual infection or a previous vaccination against *Neorickettsia risticii* (causative agent of Potomac horse fever). Therefore, immunological results alone without PCR-based confirmation need to be considered with caution [10], [11]. At the same time, screening of digeneans for *Neorickettsia* has been limited, which prevents understanding of the actual diversity of these bacteria and potential sources of infection of vertebrate animals including humans. Only 21–22 digenean species, in most cases identified only to genus or family level, have been previously confirmed as hosts of *Neorickettsia* [3], [4], [12], [13], [14], [15]. Records resulting from PCR detection of neorickettsial DNA are known only from North and South America, eastern Asia, and Antarctica. However, most of these records are based on *Neorickettsia* detected in vertebrate host tissues (horses, dogs, humans, fish). As posited by Vaughan et al. [4], future studies focusing on screening digenean extracts for *Neorickettsia* will reveal additional host associations of these bacteria and new pathways of their circulation in nature. This has already been demonstrated in a recent study by Tkach et al. [3] who found 4 species level genetic lineages of *Neorickettsia* in 7 species of digeneans belonging to 7 different families.

Currently, there are 3 named species and 10 not formally named genotypes of *Neorickettsia* that are likely to represent additional species based on levels of 16S rRNA sequence divergence [2], [3], [4], [5], [16], [17], [18], [19]. Of these 13 known species/genotypes of *Neorickettsia*, 6 have been found in North America. Circulation of digenean hosts of known *Neorickettsia* occurs in either freshwater (Rainbow trout agent, Catfish agent 1, Catfish agent 2), freshwater/terrestrial (*N*. *helminthoeca*, *N. risticii*, Elokomin Fluke Fever agent, *Diplostomum* agent), or fully terrestrial (*N. risticii*) environments [3], [4], [12], [13], [20], [21], [22]. Presently, however, there are no records of *Neorickettsia* from digeneans having fully marine life cycles. Presence of *Neorickettsia* in tissues of marine fishes in Antarctica and the Gulf of Mexico [5], [23] may suggest a marine circulation pathway, however, these authors did not screen any digeneans.

In this study, we used real-time PCR-based detection methods to study the diversity of *Neorickettsia* in previously understudied or unstudied regions. To accomplish this goal, we screened for *Neorickettsia* numerous DNA extracts from an extensive collection of adult digeneans collected from various vertebrates as well as snail hosts in the United States (several states), Australia, and China. As a result, we have found 7 new genetic lineages of *Neorickettsia* some of which may potentially represent new species. All of our records represent new digenean host associations. Our findings also expand the range of circulation pathways known for *Neorickettsia*. We have for the first time detected *Neorickettsia* from Australia and China using PCR-based detection and DNA sequencing. We have also conducted a molecular phylogenetic analysis in order to estimate interrelationships among the newly discovered genotypes with previously known named species and not yet named lineages of *Neorickettsia*.

## Materials and Methods

### Ethics statement

Digeneans were collected from marine and freshwater fishes, amphibians, reptiles, birds, mammals, and invertebrates from 2009 through 2012 from multiple localities in China, Australia, Argentina, Costa Rica, and several states in the USA (Mississippi, Louisiana, Florida, Oregon, North Dakota, Minnesota). An IACUC protocol 10100105 was issued by the University of Southern Mississippi for collecting and humane euthanasia of wild animals in Mississippi, Florida, and Louisiana. Birds were collected by shotgun in accordance to the state and federal permits, raccoons were live trapped and euthanized in the field by firearm (.22 caliber rifle) in accordance to issued permits, and amphibians and reptiles were collected by hand and humanly euthanized by immersion in a solution of chlorobutanol (chloretone) in water in accordance with the issued permits and IACUC protocol. IACUC protocols were not required for invertebrate collecting in Oregon, North Dakota, and Minnesota or for fish. Fish were purchased dead from food markets in Argentina, Costa Rica, and China. In Australia, fish were caught by cast net by one of the authors (EP) from around Townsville (Queensland), Darwin (Northern Territory) and Broome (Western Australia). Fish were placed on ice after capture, and if necessary were sacrificed with the use of a 250 mg/l bath of Tricaine methane sulfonate (MS-222) in accordance with the issued permits.

### Sample collections

All digenean samples were collected over a period of 7 years as parts of several independent projects dealing with parasite biodiversity and systematics. Thus, the source of DNA available for *Neorickettsia* screening was opportunistic, but as inclusive as possible. Required scientific collecting permits were obtained in all cases. The Florida Fish and Wildlife Conservation Commission issued the permits LSSC-11-00074 and FNW-13-05(renewal) that allowed for the collection of invertebrates, amphibians, reptiles, and mammals as well as freshwater fishes in Florida. The Mississippi Department of Wildlife Fisheries and Parks issued a permit 0123131 for the collection of invertebrates, amphibians, reptiles, mammals, and freshwater and marine fishes in Mississippi, and the Louisiana Wildlife and Fisheries issued a permit LNHP-13-017 also for the collection of the above mentioned animals in Louisiana. Additionally, a federal permit (MB681207-0) was issued by the US Fish and wildlife service for collection of reptiles, mammals, birds and freshwater fish in Mississippi and Louisiana. Three separate permits were issued in Australia for the collection of fish; Western Australia (Government of Western Australia, Department of Fisheries, SPA-01-10); Northern Territory (Northern Territory Government, Department of Resources, S17/2932); and Queensland (Department of Primary Industries and Fisheries 133621). Only abundant commercial fish species were purchased from local markets and fishermen in China, Argentina, and Costa Rica, and therefore, permits were not required. Additionally, aquatic snails were collected from North Dakota, Minnesota, and Oregon. Snails were identified using Burch's “North American Freshwater snails”[24], and also did not require permits. We do not provide a complete list of screened digenean species because in the case of *Neorickettsia* a negative result does not necessarily mean that a certain species of digenean cannot be a host for *Neorickettsia*. As discussed by Tkach et al. [3] digeneans do not have any known co-dependency with their *Neorickettsia* endosymbionts and therefore, different individuals of the same digenean species may or may not be infected.

### Sample processing

Live worms were rinsed in saline, examined briefly, killed with hot water, and fixed in 70% ethanol that allowed for both morphological examination and molecular study. When numerous specimens were found, some were fixed in 95% ethanol for molecular work. Fixed worms were stained in aqueous alum carmine, Mayer's hematoxylin, or Van Cleave's hematoxylin; dehydrated in a graded ethanol series; cleared in clove oil (carmine and Van Cleave's) or methyl salicylate (Mayer's); and mounted permanently in Damar Gum for morphological identification.

Genomic DNA was extracted from individual adult worms and pooled larval stages either using the Qiagen DNAeasy tissue kit (Qiagen, Inc., Valencia, California) following the manufacturer's instructions or the guanidine thiocyanate method according to Tkach and Pawlowski [25].

### Molecular screening

DNA extracts were first tested for the presence of *Neorickettsia* using a real-time PCR protocol designed and extensively tested by one of the authors (SEG). Five microliters of each DNA extract were used. The real-time PCR amplified a 152-bp portion of the 3′ end of the heat shock protein coding gene, GroEL. The primer pair (designed by SEG) used is listed in Table 1. Samples that tested positive with real-time PCR were verified using a substantially modified nested PCR protocol initially described by Barlough et al.[26]. Five microliters of each DNA extract were used for the first PCR reaction and 1 µl of the first PCR product was used for the nested PCR. A 1470 bp long fragment of the 16S rRNA gene was first amplified using the primer pair listed in Table 1,designed by SEG. The nested PCR step amplified a 1371-bp fragment using internal primers also listed in Table 1. The same nested PCR primers were used in sequencing reactions along with internal forward and internal reverse primers (Table 1), designed by SEG.

**Table 1:** PCR Primers used in the study.

Reaction type	Primer	Sequence (5′-3′)
Real-time	groel-1500F	ATAGATCCAGCKAAGGTAGTGCGTGT
	groel-1620R	TTCCACCCATGCCACCACCAGGCATCATTG
1^st^ round PCR (*Neorickettsia*)	n16S-25F	TCAGAACGAACGCTAGCGGT
	n16S-1500R	AAAGGAGGTAATCCAGCCGCAGGTTCAC
Nested PCR/sequencing (*Neorickettsia*)	n16s-50F	TAGGCTTAACACATGCAAGTCGAACG
	n16S-1400R	CGGTTAGCTCACTAGCTTCGAGTAA
Sequencing (*Neorickettsia*)	16S-n900F	GACTCGCACAAGCGGTGGAGTAT
	16S-n900R	ATACTCCACCGCTTGTGCGAGTC
PCR/sequencing (digenean)	digl2	AAGCATATCACTAAGCGG
	1500R	GCTATCCTGAGGGAAACTTCG
Sequencing (digenean)	300F	CAAGTACCGTGAGGGAAAGTTG
	900F	CCGTCTTGAAACACGGACCAAG
	300R	CAACTTTCCCTCACGGTACTTG
	ECD2	CTTGGTCCGTGTTTCAAGACGGG

The real-time PCR reactions were run on a Bio-Rad CFX96 Touch real-time PCR detection system (Bio-Rad Laboratories, Hercules, CA) using iTaq universal sybr green supermix (Bio-Rad Laboratories, Hercules, CA) according to the manufacturer's instructions. A two-step program was used with a denaturation temperature of 95°C for 3 seconds, followed by an annealing and extension temperature of 64°C for 25 seconds and 36 cycles. In addition, a melt curve was run starting from 72°C and increasing at 0.2°C increments every 5 seconds until reaching 87°C. The nested PCR reactions were run on EP Gradient thermocycler (Eppendorf, Hauppauge, NY) using Quick load OneTaq mastermix (New England Biolabs, Ipswich, MA) according to the manufacturer's instructions. Annealing temperature of 56°C and 40 cycles were used in both first and nested PCRs. DNA of *N. sennetsu* used as a positive control was graciously provided by Dr. Sabine Dittrich (Lao Oxford Mahosot Wellcome Trust Research Unit). Pure water was used for negative controls in both real-time and nested PCRs.

### Digenean host identification

Adult digeneans were identified based on their morphology using stained whole mounts. Identification to generic level was done using the three-volume “Keys to the Trematoda” [27] [28] [29] with subsequent species identification done using numerous original descriptions and revision articles. *Neorickettsia* positive cercariae or metacercariae were identified to the lowest possible taxonomic level using a partial sequence of the nuclear large ribosomal subunit gene (28S). Digenean DNA was amplified from the extracted DNA by PCR using the primer pair listed in Table 1. The same PCR primers and additional internal primers were used for sequencing (Table 1).

### DNA sequencing

PCR amplicons of both *Neorickettsia* and digeneans were purified using the Zymo DNA Clean & Concentrator -5(Zymo Research, Irvine, CA) or ExoSap PCR clean-up enzymatic kit from Affimetrix (Santa Clara, CA) according to the manufacturer's instructions. The PCR products were cycle-sequenced using ABI BigDye chemistry, ethanol precipitated, and run on an ABI Prism 3100 automated capillary sequencer. Contiguous sequences of *Neorickettsia* and digeneans were assembled using Sequencher ver. 4.2 (GeneCodes Corp., Ann Arbor, MI) and submitted to GenBank under accession numbers KF661342-KF661351 and KF878083- KF878084.

### Phylogenetic analysis

**Figure 1:**
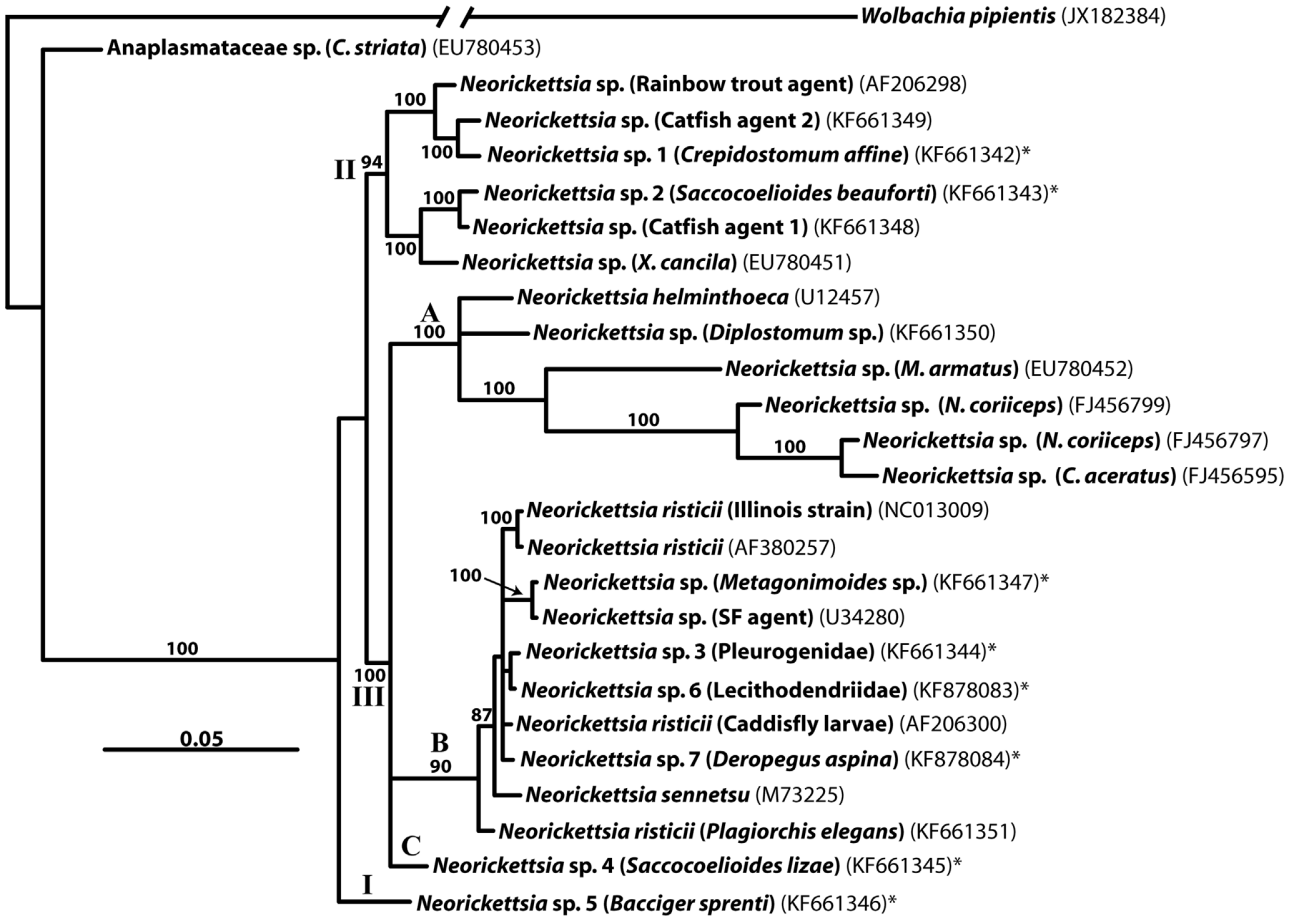
Phylogenetic relationships among 25 taxa of bacterial endosymbionts in the family Anaplasmataceae resulting from Bayesian analysis. Phylogenetic relationships among 25 taxa of bacterial endosymbionts in the family Anaplasmataceae resulting from Bayesian analysis (1,500,000 generations) of partial sequences of 16S rDNA gene. Posterior probabilities greater than 80% are shown above internodes. Roman numerals (I, II, II) represent the different clades within the “*Neorickettsia* clade” and letters (A, B, C) correspond to the subclades within clade III. An asterisk (*) at the end of a taxon name indicates a new genotype of *Neorickettsia* discovered in this study. GenBank numbers are given here for all taxa.

Newly obtained sequences (Table 2 and Fig. 1) and sequences of neorickettsiae from GenBank were used in the phylogenetic analysis. The new sequences and sequences obtained from GenBank were initially aligned with the aid of ClustalW as implemented in the BioEdit program, version 7.0.1 [30]. The alignments were manually refined in MacClade, version 4 [31].

**Table 2:** *Neorickettsia* genotypes, natural hosts, types of life cycles, life cycle stage, geographic origins, definitive and intermediate hosts.

Neorickettsia species	Digenean family	Digenean genus and species	Life cycle stage	Host (this study)	Definitive host	1^st^/2^nd^ intermediate hosts	Life cycle	Locality
*Neorickettsia* sp. 1 (KF661342)	Allocreadiidae	*Crepidostomum affine*	Adult	Mooneye (*Hiodon tergisus*)	Fishes	Unknown/aquatic arthropod	aquatic (freshwater)	Pearl River, Mississippi
*Neorickettsia* sp. 2 (KF661343)	Haploporidae	*Saccocoelioides beauforti*	Adult	Striped mullet (*Mugil cephalus*)	Fishes	unknown	aquatic (brackish/marine)	Cedar Key, Florida
*Neorickettsia* sp. 3 (KF661344)	Pleurogenidae	Unknown	Metacercariae	Crayfish (*Procambarus* sp.)	Mammals and amphibians	Aquatic snail/crustacean	aquatic/terrestrial (freshwater)	Williford Springs Florida
SF agent (KF661347)	Heterophyidae	*Metagonimoides oregonensis*	Adult	Raccoon (*Procyon lotor*)	Mammals	Aquatic snail/amphibian	aquatic/terrestrial (freshwater)	Williford Springs Florida
*Neorickettsia* sp. 4 (KF661345)	Haploporidae	*Saccocoelioides lizae*	Adult	Striped mullet (*Mugil cephalus*)	Fishes	Unknown	aquatic (brackish/marine)	Daya Bay, Guangdong, China
*Neorickettsia* sp. 5 (KF661346)	Faustulidae	*Bacciger sprenti*	Adult	Spotbanded scat (*Selenotoca multifasciata*)	Fishes	Unknown	aquatic (brackish/marine)	Eli Creek, Queensland, Australia
*Neorickettsia* sp. 6 (KF878083)	Lecithodendriidae	*Prosthodendrium* sp.	Cercariae	Stream snail (*Juga yrekaensis*)	Bats and birds	Aquatic snail/aquatic arthropod	aquatic/terrestrial (freshwater)	Dunn Forest, Corvallis, Oregon
*Neorickettsia* sp. 7 (KF878084)	Derogenidae	*Deropegus aspina*	Cercariae	Stream snail (*Juga yrekaensis*)	Fishes and amphibians	Aquatic snail/aquatic arthropod	aquatic/terrestrial (freshwater)	Dunn Forest, Corvallis, Oregon

Phylogenetic analysis was carried out using Bayesian inference (BI) as implemented in the MrBayes program, version 2.01 [32] with the following nucleotide substitution parameters: lset nst = 6, rates = invgamma, ngammacat = 4, that correspond to a general time reversible (GTR) model including estimates of the proportion of invariant sites (I) and gamma (G) distributed among-site rate variation. Posterior probabilities were approximated over 1,500,000 generations for both data-sets, log-likelihood scores plotted and only the final 75% of trees were used to produce the consensus trees by setting the “burnin” parameters at 375,000 generations. The GTR+I+G model was used for the analysis based off of the results obtained from jModelTest, version 0.1.1 [33], [34].

## Results

A total of 775 digenean samples were screened for *Neorickettsia*. A list of digenean families screened and the number of extracts corresponding to each family are provided in Table 3. Screening revealed 8 different genetic lineages of *Neorickettsia* in 8 digenean species belonging to 7 different families (Table 2). *Neorickettsia* infections were detected in the following collecting sites: Pearl River, Mississippi (30°20′36″N, 89°38′03″W); Cedar Key, Florida (29°08′13″N, 83°02′36″W); Williford Springs, Florida (30°26′20.40″N, 85°32′50.77″W and 30° 26′21.95″N, 85°32′42.26″W); Daya Bay, Guangdong, China (22°43′N 114°32′E); Eli Creek, Queensland, Australia (25°15′45″S, 152°48′28″E). We have detected *Neorickettsia* from digeneans for the first time from the Australian continent and China as well as from Oregon and Florida. One of the forms discovered in our study is identical to the SF agent (Table 2). Sequences of the 7 other forms clearly differed from all other known forms of *Neorickettsia*, therefore, potentially representing new species.

**Table 3:** List of digenean families screened for *Neorickettsia* and number of individual extracts associated with each family.

Digenean Family	Number of extracts	Digenean Family	Number of extracts	Digenean Family	Number of extracts
Acanthocolpidae	2	Fellodistomoidae	3	Paramphistomidae	2
Allocreadiidae	40	Gorgoderidae	33	Philophthalmidae	5
Apocreadiidae	46	Haploporidae	227	Pleurogenidae	4
Atracotrematidae	4	Haplosplanchnidae	17	Pronocephalidae	4
Azygiidae	13	Hemiuridae	6	Psilostomidae	4
Bivesculidae	8	Heterophyidae	5	Renicolidae	2
Bucephalidae	54	Lecithodendriidae	17	Rhopaliidae	2
Clinostomatidae	3	Lepocreadiidae	43	Spirorchiidae	8
Cryptogonimidae	20	Lissorchiidae	8	Telorchiidae	4
Cyathocotylidae	2	Macroderoididae	23	Troglotrematidae	8
Derogenidae	6	Microphallidae	28	Zoogonidae	3
Diplostomatidae	16	Monorchiidae	17	Unidentified	30
Echinostomatidae	1	Notocotylidae	1		
Eucotylidae	1	Opecoelidae	22		
Faustulidae	22	Opisthorchiidae	7		

**Figure 2:**
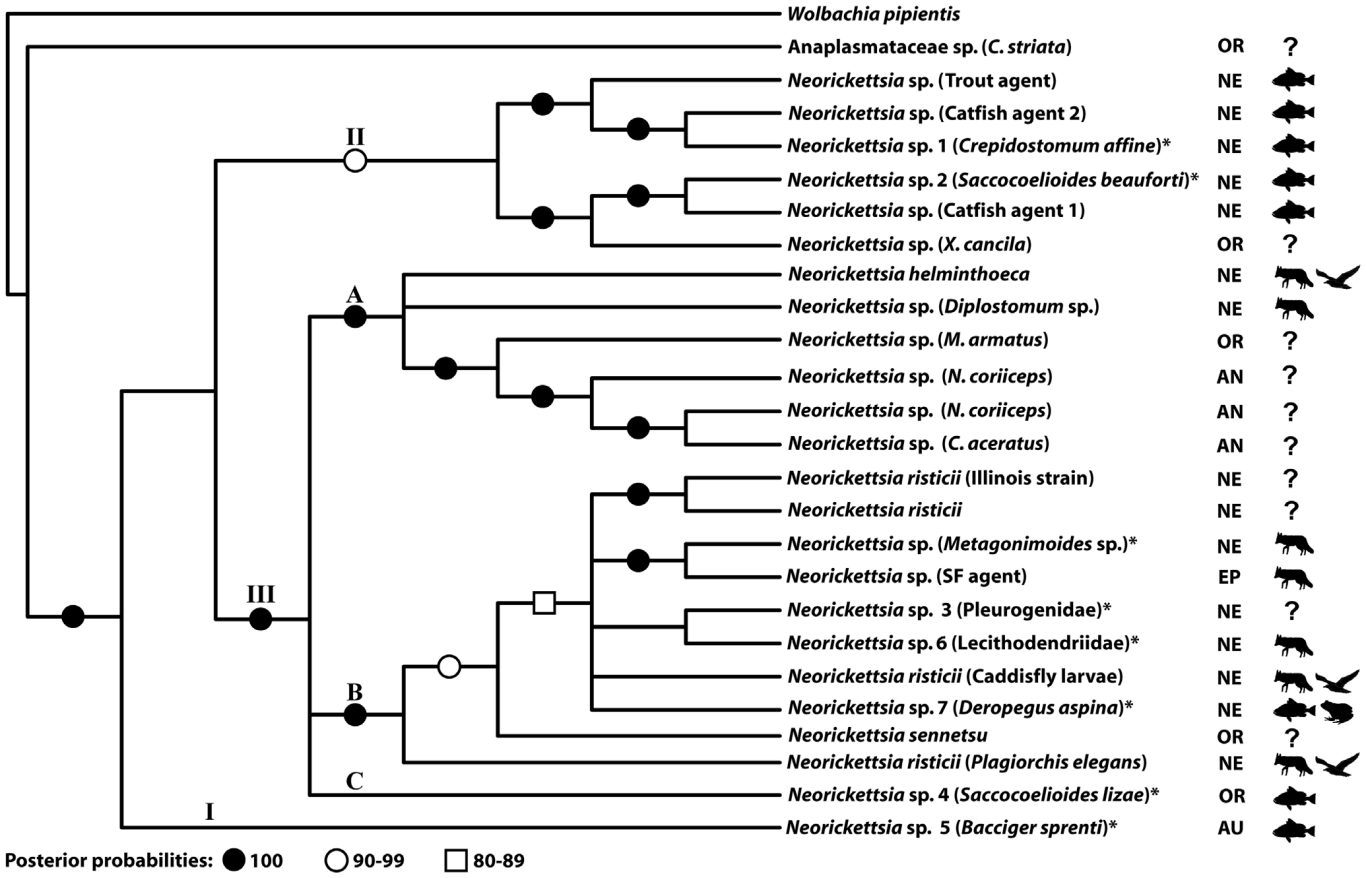
Phylogenetic tree resulting from Bayesian analysis (1,500,000 generations) of partial sequences of 16S rDNA gene. Phylogenetic tree resulting from Bayesian analysis (1,500,000 generations) of partial sequences of 16S rDNA gene. Posterior probabilities greater than 80% are shown. Roman numerals (I, II, II) represent the different clades within the “*Neorickettsia* clade” and letters (A, B, C) correspond to the subclades within clade III. An asterisk (*) at the end of a taxon name indicates a new genotype of *Neorickettsia* discovered in this study. Digenean vertebrate definitive host groups (fishes, mammals and birds) are indicated by symbols. Zoogeographic regions are shown as the following; NE (nearctic), EP (eastern palearctic), OR (oriental), AU (australian), and AN (antarctic).

The phylogentic analysis was run using a dataset including 25 ingroup taxa and *Wolbachia pipientis* (a member of Anaplasmataceae endosymbiotic in insects and filariid nematodes) as an outgroup. The alignment included a total of 1,258 sites, of which 1,249 could be aligned unambiguously. Positions that could not be aligned unambiguously were excluded from the analysis. Bayesian analysis produced a tree where all *Neorickettsia* sequences clearly fall into a well-defined clade, with a 100% support (Figs. 1 and 2).

The “*Neorickettsia* clade” includes three major, well supported clades (Figs. 1 and 2). Clade I is represented by a single sequence from *Bacciger sprenti* in Australia. Clade II includes 6 sequences, all of them representing yet unnamed lineages/species of *Neorickettsia* found in either digeneans parasitic in fish or in fish tissues. Two of these forms are represented by our newly sequenced genotypes, *Neorickettsia* sp. 1 (from *Crepidostomum affine* in Mississippi) and *Neorickettsia* sp. 2 (from *Saccocoeliodes beauforti* from Florida). All internal sub-clades of clade II are well resolved and 100% supported. Five lineages of this group are from North America while one is from Southeast Asia.

The major clade III contains a majority of the *Neorickettsia* species, including all three currently recognized named species of these bacteria, namely *N. helminthoeca*, *N. risticii*, and *N.sennetsu* (Figs. 1 and 2). This large clade is split into three sub-clades indicated as A, B, C on Figures 1, 2. Sub-clade A contains a diverse assemblage of lineages that includes *N. helminthoeca*, *Neorickettsia* sp. from a *Diplostomum* sp. published by Tkach et al. [3] as separate branches, and a well resolved cluster containing one form from Southeast Asia and three genotypes from the Antarctic [5]. All digenean hosts of the members of this sub-clade are parasitic in fish at least at some phase of their life cycle. Sub-clade B is 100% supported, however, internal interrelationships of its constituent taxa are less resolved than most of the topologies elsewhere in the tree. This sub-clade incorporates 4 *Neorickettsia* forms discovered in this study. The basal taxon in this sub-clade is the form reported as *Neorickettsia risticii* by Tkach et al. [3]. The closest derived taxon to it is *N. sennetsu*, the agent causing human disease in Southeast Asia. The remaining lineages of the sub-clade B form a polytomy that includes several lineages of *N. risticii* and the SF agent. Sub-clade C contains only our newly sequenced form from China. All species/genotypes of *Neorickettsia* in this clade are associated with digeneans who use mammals and/or birds as their definitive hosts.

## Discussion

The newly developed real-time PCR protocol has provided for a quick and sensitive way of screening large collections of digenean DNA extracts for *Neorickettsia*. Removing the necessity of running a nested PCR and screening the results with gel electrophoreses for every extract has greatly reduced the amount of time required to screen large collections of DNA extracts. The elimination of the use of post-PCR handling steps in real-time PCR also reduces the likelihood of false positive results caused by contamination. Although real-time PCR using TaqMan probes has been used in the detection of *N. risticii* from snails and horses [35], the technique was not used for a broad screening of digeneans for *Neorickettsia* and there was no published protocol of “regular” real-time PCR using sybr green or another dye as an alternative to the TaqMan probes. Another advantage of the real-time PCR protocol is the ability to run melting curve analysis, which is particularly useful for differentiating potential non-specific binding of primers. It was of great importance for our study since we used relatively generic primers targeting all *Neorickettsia* species and genotypes. The combination of real-time PCR, nested PCR, and sequencing allows for a very robust and accurate screening.

Our screening has revealed 8 different genotypes of *Neorickettsia*: a new genotype 1 from Mississippi, new genotypes 2, 3, and SF agent from Florida, new genotypes 6 and 7 from Oregon, a new genotype 4 from China, and a new genotype 5 from Australia (Table 2). Our findings represent the first records of *Neorickettsia* in digeneans in Australia, China, and two states (Oregon and Florida) in the USA. All 8 digenean species detected as hosts of *Neorickettsia* in our study (Table 2) represent new host associations for *Neorickettsia*, although Pusterla et al. [13] detected *Neorickettsia* within a species of *Deropegus* from California, neither the species of *Deropegus* or *Neorickettsia* were identified or sequenced. Members of the families Haploporidae, Pleurogenidae, and Faustulidae have not been previously reported as hosts of *Neorickettsia*.

Sampling of digenean DNA extracts for *Neorickettsia* was mostly opportunistic. As stated in the Materials and Methods, digeneans were collected for various projects prior to our study and were not initially intended for detecting *Neorickettsia*. This explains the unevenness in the distribution of screened samples among digenean families. Digeneans were not targeted based on their likelihood of harboring the bacterial endosymbionts because very little is currently known about the evolutionary associations among *Neorickettsia* and their digenean hosts. Therefore, we screened as many samples and as diverse digenean taxa as possible. Our study is the first geographically and taxonomically broad screening of digeneans for the presence of *Neorickettsia*. Its purpose is to provide baseline data for future more focused studies within certain digenean families or those parasitic in certain hosts.

Our study has revealed a new circulation pathway of *Neorickettsia* in the natural environment. Until now, *Neorickettsia* have been found in digeneans with entirely freshwater, freshwater/terrestrial or entirely terrestrial life cycles [3], [4], but not in digeneans with completely marine life cycles. Reports of *Neorickettsia* in tissues of notothenioid fishes (*Notothenia coriiceps* and *Chaenocephalus aceratus*) in Antarctica [5] and mullet *Mugil cephalus* in the Gulf of Mexico [23] may suggest a marine circulation pathway, however, neither of these studies screened any digeneans. We have found three different forms of *Neorickettsia* (genotypes 2, 4, and 5) from three digenean species with completely marine life cycles, namely *Saccocoeliodes beauforti*, *Saccocoelioides lizae*, and *Bacciger sprenti*. This suggests that *Neorickettsia* can be encountered in almost every type of environment suitable for digenean life cycles.

One of our *Neorickettsia* genotypes obtained from *Metagonimoides oregonensis* in Florida fully matched the sequence of SF agent. SF agent was initially found in metacercariae of the heterophyid *Stellantchasmus falcatus* [36] infecting grey mullet in Japan [13], [37]. Our finding is the first record of SF agent outside Japan. Both *S. falcatus* and *M. oregonensis* belong to the family Heterophyidae, therefore, there is a probability that SF agent could be found in other members of this large digenean family, members of which are parasitic primarily in fish-eating birds and mammals including humans. SF agent causes mild clinical symptoms in experimentally infected mice and dogs, but not monkeys or humans [8], [11], [38], [39], [40], [41]. Thus, it cannot be excluded that it may cause yet unknown disease in wildlife.

Our phylogenetic analysis has produced a tree with strong branch support for most topologies (Figs. 1 and 2). Our data corroborate the conclusion by Seng et al. [2] who suggested that their sequence obtained from *Channa striata* most likely represents a separate genus of Anaplasmataceae. On the other hand, the Anaplasmataceae sp. from *Mastacembelus armatus* considered by Seng et al. [2] to be another new genus, clearly falls into the clade II of the “*Neorickettsia* clade”(Figs 1, 2). *Neorickettsia* sp. 5 genotype is of particular interest because it represents a separate clade of *Neorickettsia* with unresolved affinities to other members of the genus. Denser sampling of digeneans from both marine and freshwater habitats in Australia is necessary to see whether *Neorickettsia* sp. 5 is a unique, divergent form or a member of a larger lineage that includes additional, yet undiscovered, taxa. An additional systematically relevant result of the present phylogenetic analysis is the position of the *N. risticii* strain from *Plagiorchis elegans*. This form was initially identified by Tkach et al. [3] as *N. risticii* based on the comparison of much shorter DNA sequences and a phylogenetic analysis using those shorter sequences and fewer taxa. In the analysis by Tkach et al. [3] this genotype appeared in a polytomy that included 4 different isolates of *N. risticii* and *N. sennetsu*. The present analysis based on longer sequences and greater number of taxa places “*N. risticii*” from *P. elegans* as the basal lineage in sub-clade B of clade III. It is separated from the remaining taxa in this clade by one of the recognized distinct species, *N. sennetsu*. Thus, this genotype likely represents a different, novel species of *Neorickettsia* which needs to be better characterized and differentiated from formally named taxa.

The least resolved part of the tree is the polytomy in sub-clade B of clade III that includes several genotypes occupying a derived position in relation to *N. sennetsu* (Figs. 1, 2). The relationships between these genotypes are likely to be clarified with the inclusion of additional genes in future analyses. The last systematic consideration stemming from our analysis is the unresolved position of *N. helminthoeca* in relation to other members of the sub-clade A of clade III. Unfortunately, the only 16S sequence of *N. helminthoeca* available in the GenBank is of poor quality and contains numerous unresolved/problematic positions. We believe that obtaining new high quality sequences of *N. helminthoeca* will help to clarify its affinities with the other species/genotypes of *Neorickettsia*.

Some of the clades in the phylogenetic tree show close associations among *Neorickettsia* and the definitive hosts of digeneans in which they were found. For instance, the strongly supported clade II notably comprises neorickettsiae obtained from digeneans parasitic only in fish, mostly in North America. This clade shows the most clear association with a group of definitive hosts of digeneans. Another group that demonstrates a distinct pattern of associations with definitive hosts of digeneans is the sub-clade B of clade III. Digenean hosts of neorickettsiae in this sub-clade are parasitic in either birds or mammals with the exception of few cases where the vertebrate hosts of digeneans are unknown and *Neorickettsia* sp. 7 found in *Deropegus aspina*, a digenean that use fish and amphibians as a definitive host (Fig. 2). *N. sennetsu*, a causative agent of the human disease Sennetsu fever in Southeast Asia, is one of the species with yet unknown digenean hosts. Nevertheless, the phylogenetic tree topology allows us to hypothesize that it should be a digenean that uses either mammals or birds as a definitive host.

Patterns of geographic distribution of most of the digenean species harboring *Neorickettsia* in this study are varying and in some cases little is known. *Crepidostomum affine* was recently described by Tkach et al. [42] from *Hiodon tergisus* collected from Pearl River and Pascagoula River drainages in Mississippi, USA. Based on a molecular comparison to other species in the genus by Tkach et al. [42], *C. affine* is likely endemic to these river basins in Mississippi. Therefore, we hypothesize that the distribution of *Neorickettsia* sp. 1 is likely to be limited to that of *C. affine*.

*Mugil cephalus*, the host of two digenean species harboring *Neorickettsia* in this study (Table 2), is a globally distributed marine fish. However, these two digenean species, *Saccocoelioides beauforti* and *Saccocoelioides lizae*, are much more restricted in their distribution than their fish host. One of them, *Saccocoelioides beauforti* has only been reported from the southeastern United States (North Carolina, Louisiana, Mississippi, and Alabama) [43], [44]. The other species, *Saccocoelioides lizae* has only been found off the cost of South-eastern China [45]. Based on the combined distribution data of these digeneans and their fish host, it is likely that *Neorickettsia* sp. 2 may be found in other areas along the coast of the southeastern United States and that *Neorickettsia* sp. 4 may be found in other costal countries in Southeast Asia.

*Deropegus aspina* is only known from the Pacific Northwest of the United States in salmonid fishes, including *Salmo clarki*, *S. gairdneri*, *Oncorhynchis kisutch*, *O. tshawytscha*, and a frog, *Rana boyli*, [46]. Therefore, we hypothesize that *Neorickettsia* sp. 7 is limited in its distribution to the Pacific coast of the USA and Canada.

*Bacciger sprenti* (family Faustulidae) carrying *Neorickettsia* in our study was obtained from fish *Selenotoca multifasciata* (Table 2). This digenean species was originally identified from the intestine of a *Mugil* sp. in Australia [47]. However, later research by Cribb et al. [48] only found it in *Selenotoca multifasciata* (13 individuals were infected) and not in 11 individuals of *Mugil cephalus* or 49 individuals of *Mugil georgii*. This led Cribb et al. [48] to speculate that an error was made in the recording of the host fish species in the original description by Bray [47]. This is in concordance with available data on host associations of other faustulid trematodes, which are not known to be shared between scatophagid and mugilid fishes [48]. Currently *Bacciger sprenti* has only been reported from marine fishes in Australian coastal waters, therefore, we can assume *Neorickettsia* sp. 5 is a genotype unique to Australia.

Currently known distribution of *Neorickettsia* is very geographically uneven and is clearly associated with areas where most studies have taken place. The majority of genotypes were found in the USA and several countries in southeastern and eastern Asia [4]. Even after our discovery of *Neorickettsia* in Australia, there is no well-documented information supported by PCR/sequence data from Africa, Europe, most of South America, most of Asia, and nearly all island countries [4]. We believe that this situation does not reflect the true distribution of *Neorickettsia* and rather reflects insufficient knowledge due to the lack of broad screening efforts. With more than 18,000 described species of digeneans [49], there is a potential for many more species/genotypes of *Neorickettsia* to be found and characterized.
